# The American Dental Association

**Published:** 1884-08

**Authors:** 


### THE AMERICAN DENTAL ASSOCIATION.

On the fifth of August, the twenty-fourth annual meeting of the A. D. A. will commence its sessions in the Supreme Court Chamber of the Town Hall at Saratoga. In some essential respects this is the most important of all our societies, and its meetings should be attended by every representative man of the profession. That the society is all that its friends would be glad to have it, no one pretends. But it cannot well be made better unless there is a more general attendance upon its meetings. It is, perhaps, impossible that any one society should comprise within its membership all the intelligence of the profession. But the representative association of dentistry should command a membership and attendance commensurate with its assumed position. If the American Dental Association cannot challenge the confidence and support of the profession, we shall heartily join in any movement that promises anything better. But that has not as yet been demonstrated. There has been at times a lack of enthusiasm, and the society has been handicapped too heavily. There has been a lack of carefully digested papers, and the discussions have sometimes been trivial. But the A. D. A. has a glorious record, and it only remains for the members to awaken to the importance of the occasion, and to remember that such an annual meeting is intended for something else than the consideration of local subjects, or the strife for position that has sometimes been observed. The men who will meet at Saratoga are abundantly competent to grapple with the broad principles that underlie dental Science, and from the mutual interchange of convictions and experience to educe rules of practice that shall be a law unto dentistry. But to accomplish this, mere considerations of personality, narrow prejudices in favor of individual methods, and preconceived opinions founded upon a limited observation should all be laid aside, and members should meet, resolved by careful deliberations to determine what in any particular case is most in accord with an enlightened knowledge of scientific practice.

The main object that should be kept steadily in view in such convocations is the discussion of scientific problems. The business questions, the machinery of the society, demand attention, but the constant tinkering with the by-laws, the endless introduction of amendments, and the long-winded, tiresome debates upon questions of order and points of precedence, are depressing in their influence. Thinking men do not travel hundreds of miles to discuss parliamentary laws. They desire enlightenment upon principles of practice. They wish to exchange views upon matters that pertain to their every-day business. If the President is prompt in his rulings, there is little necessity for hair-splitting concerning the exactness of his decisions. Even by-laws may at times, with propriety, yield to the best interests of the whole. It is to be earnestly hoped that every member of the A. D. A. will be present at the coming meeting, and that all will be dominated by the desire to make of it a memorable time, an anniversary long to be remembered for the profit that each shall derive from it.

At the close of the sessions, on Friday evening, will be held the meeting of the National Association of Dental Examiners. There are many questions of importance that should come before this body, and all State Boards are earnestly requested to send delegates.

